# Mortality of Philadelphia, for July, August and September, 1856; Collated from the Record at the Health Office

**Published:** 1856-11

**Authors:** Wilson Jewell


*Mortality of Philadelphia, for July, August and September,* 1856*; collated from the Record at the Health Office.* By Wilson Jewell, M. D.

The whole mortality of the city on record at the Health Office, for the third quarter of the year, and ending Sept. 27th, amounts to 3971. An increase of 7·83 per cent. over the same period of 1855, when the deaths stood 3387.

The mortality from diseases, so recorded, amounted to 3408. Of these, 1522, or more than 44 per cent. are arranged under the head of Epidemics, and are the result of removable diseases, the existence of which, in all large cities, have been ascribed, and not unaptly so, to a defective sanitary system. The remaining deaths on record, numbering 563, were occasioned by External Causes, Old Age, Still-Born, Debility and Unknown.

The excess of deaths in the sexes, according to the aggregate, is among the males, amounting to 6·09 per cent.

The excess of deaths in males from External Causes amounts to 90, or 51·72 per cent. This alone, constitutes more than one-third the entire excess, which has been attributed to the greater exposure of males ; in the case of children, however, this explanation is not received as satisfactory.

Within the first year of life, exclusive of Still-Born, the deaths amounted to 1246, or 31·37 per cent. of the total. While within the 5th year, they were 2309, or 58·12 per cent.

Under twenty years of age there were 2784 deaths, equal to 67·59 per cent. of the mortality for the quarter.

Between twenty and eighty, they numbered 1150, and beyond eighty, 56, two of which were centennarians.

The highest number of deaths per week was 426, which occurred between the 19th and 26th of July. This month, July, contributed 1796 deaths, while September gave only 864, a falling off of more than one-half.

The deaths from the Endemic class of diseases amounted to 1522, or nearly one-half of the deaths from certified diseases. The third quarter of the year usually exhibits a large proportion of deaths from this division of diseases. Cholera Infantum alone contributes 676 ; while Dysentery gives 232 ; Scarlet Fever 179, and Measles 69. The two last named have been widely prevalent during the quarter, especially Measles. Last year, in the same period, there were only 26 deaths from Scarlet Fever, while from Measles, not a death was reported.

The mortality from fevers amounted to 295, an increase of more than 31 per cent. over those for the same period of 1855. The increase has been caused chiefly by the increased prevalence of Scarlet Fever and Measles over that of last year.

Four deaths from Typhus Icterodes are recorded. Three of these cases we had an opportunity of witnessing, and have no hesitation in believing them to have been genuine instances of Sporadic Yellow Fever. Two of them died in Kensington district ; their residence was in the same house and about two blocks from the river Delaware. This happened in July, at that period when the reservoir water used by the citizens for drinking and other purposes, was in a fœtid condition, owing to the decomposition of vegetable and animal matter which had accumulated in the basin.

The third case died at Cape Island, N. J. This young man was engaged in a counting house on Delaware avenue, below Race st. He sickened on the evening of his arrival at Cape Island, died on the eighth day from attack and was brought to the city for interment. None of these persons had been on board of any vessel from the West Indies that had been infected from yellow fever. Nor could we learn that any other persons had labored under the disease, except a young woman, who resided with the Kensington family, whose case occurred at the same time with the other two, but was of so mild a type as to render its true character doubtful. She was removed from the atmosphere of the house to the City hospital, and recovered after a week's sickness, the fever yielding on the third day, when her system reacted, followed by rapid convalescence.

The origin of these cases is involved in obscurity, and until otherwise informed we must attribute them to indigenous causes.

Of the "Uncertain or General Seat," there were 605 deaths. An increase over those for last year of 122. Marasmus, Debility and Dropsy make up 50 per cent. of the whole number.

Of the 624 deaths from diseases of the Nervous System, 73 per cent. were in children under five years of age. One hundred and eighty-one from Convulsions and one hundred and eleven from Inflammation of the Brain.

The organs of Respiration furnished 515 deaths. Of this number 370, about 71 per cent., were from Consumption of the Lungs. This number makes the deaths from Consumption 11 per cent. more than those for the same period of last year.

Of the diseases of the "Organs of Generation," amounting only to 27, eighteen of them occurred among lying-in women. By reference also to the deaths from the Digestive Organs, it will be observed, that eight females died from Peritoneal inflammation, of which deaths, we have reason to infer, several took place in the lying-in chamber.

Of the deaths for the quarter, 185 were from the Alms House; 187 were blacks; 47 from the country; 6 from the County Prison; 1 from the E. Penitentiary, and 22 from the Pennsylvania Hospital.

TABLE NO. I.

*Deaths for the third quarter of* 1856 *classified.*

| | July | Aug | Sept | Male. J. | Male. A. | Male. S. | Female. J. | Female. A. | Female. S. | Total. |
|---|---|---|---|---|---|---|---|---|---|---|
| 1 *Endemic & Contagious diseases* Zymotic or Epidemic | 729 | 530 | 263 | 396 | 293 | 142 | 333 | 237 | 121 | 1522 |
| 2 *Uncertain or general seat,* Sporadic diseases | 233 | 211 | 161 | 110 | 102 | 79 | 123 | 109 | 82 | 605 |
| 3 Nervous system | 304 | 194 | 126 | 175 | 102 | 75 | 129 | 92 | 51 | 624 |
| 4 Organs of Respiration | 214 | 157 | 144 | 88 | 76 | 68 | 126 | 81 | 76 | 515 |
| 5 " Circulation | 31 | 14 | 10 | 16 | 5 | 3 | 15 | 9 | 7 | 55 |
| 6 Digestive organs | 66 | 57 | 45 | 40 | 33 | 17 | 26 | 24 | 28 | 168 |
| 7 Urinary " | 4 | 5 | | 4 | 3 | | | 2 | | 9 |
| 8 Organs of Generation | 13 | 9 | 5 | | | | 13 | 9 | 5 | 27 |
| 9 " Locomotion | 1 | 3 | 3 | | 1 | 2 | 1 | 2 | 1 | 7 |
| 10 Integumentary system | 2 | 5 | 4 | 2 | 4 | 2 | | 1 | 2 | 11 |
| 11 Old age | 16 | 10 | 10 | 2 | 5 | 2 | 14 | 5 | 8 | 36 |
| 12 External causes | 95 | 44 | 35 | 73 | 32 | 27 | 22 | 12 | 8 | 174 |
| Still Born | 59 | 45 | 35 | 34 | 24 | 20 | 25 | 21 | 15 | 139 |
| Unknown | 29 | 27 | 23 | 21 | 16 | 13 | 8 | 11 | 10 | 79 |
| | 1796 | 1311 | 864 | 961 | 696 | 450 | 835 | 615 | 414 | 3971 |

TABLE NO. 2.

1. *Endemic and Contagious Diseases—Zymotic or Epidemic.*

| | Male. | Female. | Under 1 yr. | 1 to 2. | 2 to 5. | 5 to 10. | 10 to 15. | 15 to 20. | 20 to 30. | 30 to 40. | 40 to 50. | 50 to 60. | 60 to 70. | 70 to 80. | 80 to 90. | 90 to 100. | 100 to 110. | Total. |
|---|---|---|---|---|---|---|---|---|---|---|---|---|---|---|---|---|---|---|
| Cholera asphyxia | 7 | 5 | 3 | 1 | | 1 | | 1 | | 1 | 1 | 3 | 1 | | | | | 12 |
| " infantum | 352 | 324 | 438 | 201 | 37 | | | | | | | | | | | | | 676 |
| " morbus | 8 | 18 | 1 | 2 | 1 | | 3 | 1 | 8 | 3 | 2 | 1 | 2 | 1 | | 1 | | 26 |
| Croup | 23 | 18 | 3 | 7 | 26 | 5 | | | | | | | | | | | | 41 |
| Diarrhœa | 44 | 54 | 47 | 21 | 3 | 1 | | | 3 | 2 | 5 | 3 | 2 | 8 | 3 | | | 98 |
| Dysentery | 149 | 83 | 42 | 45 | 31 | 32 | 4 | 7 | 16 | 6 | 13 | 13 | 9 | 10 | 3 | 1 | | 232 |
| Erysipelas | 15 | 4 | 5 | 3 | | 2 | | 1 | 2 | 3 | 1 | 1 | | 1 | | | | 19 |
| Fever, | 3 | | | | | 1 | | 1 | | | | 1 | | | | | | 3 |
| " Bilious | 5 | 4 | | | | | | 1 | 3 | 1 | 2 | | | 2 | | | | 9 |
| " Congestive | 1 | | | | | | | | 1 | | | | | | | | | 1 |
| " Intermittent | 1 | 2 | 1 | 1 | | | | | 1 | | | | | | | | | 3 |
| " Nervous | | 1 | | | 1 | | | | | | | | | | | | | 1 |
| " Remittent | 2 | 3 | 1 | 1 | 2 | | 1 | | | | | | | | | | | 5 |
| " Scarlet | 103 | 76 | 16 | 41 | 85 | 31 | 4 | | 1 | 1 | | | | | | | | 179 |
| " Typhoid | 38 | 33 | | 1 | 4 | 5 | 8 | 8 | 22 | 8 | 8 | 4 | 2 | 1 | | | | 71 |
| " Typhus | 15 | 4 | 1 | 1 | | 2 | 2 | 1 | 3 | 4 | 1 | 1 | 2 | 1 | | | | 19 |
| " " Icterodes | 3 | 1 | | | | | | 3 | | | 1 | | | | | | | 4 |
| Hooping Cough | 10 | 10 | 8 | 7 | 5 | | | | | | | | | | | | | 20 |
| Measles | 33 | 36 | 20 | 25 | 21 | 3 | | | | | | | | | | | | 69 |
| Small Pox | 16 | 12 | 12 | 3 | 3 | 4 | 1 | | 4 | | 1 | | | | | | | 28 |
| Syphilis | | 1 | | | 1 | | | | | | | | | | | | | 1 |
| Varioloid | 3 | 2 | 1 | 1 | 1 | | | | 1 | 1 | | | | | | | | 5 |
| | 831 | 691 | 599 | 361 | 221 | 87 | 23 | 24 | 65 | 30 | 35 | 27 | 18 | 24 | 6 | 2 | | 1522 |

*2. Uncertain or General Seat—Sporadic Diseases.*

| | Male. | Female. | Under 1 yr. | 1 to 2. | 2 to 5. | 5 to 10. | 10 to 15. | 15 to 20. | 20 to 30. | 30 to 40. | 40 to 50. | 50 to 60. | 60 to 70. | 70 to 80. | 80 to 90. | 90 to 100. | 100 to 110. | 110 to 120. | Total. |
|---|---|---|---|---|---|---|---|---|---|---|---|---|---|---|---|---|---|---|---|
| Abscess | 4 | 1 | 1 | | | | | 2 | 1 | | | | | 1 | | | | | 5 |
| " Psoas | 1 | | | | | | | | | 1 | | | | | | | | | 1 |
| Cachexia | 1 | 3 | 1 | | 1 | | 1 | | | 1 | | | | | | | | | 4 |
| Cancer | 4 | 19 | | | | | | | 1 | 4 | 5 | 3 | 6 | 3 | 1 | | | | 23 |
| Cyanosis | 6 | 3 | 8 | | 1 | | | | | | | | | | | | | | 9 |
| Debility | 65 | 70 | 69 | 6 | 8 | 1 | | 1 | 4 | 3 | | 8 | 16 | 13 | 2 | 3 | 1 | | 135 |
| Disease of Throat | | 1 | | | | 1 | | | | | | | | | | | | | 1 |
| Dropsy | 27 | 37 | 2 | 1 | 10 | 7 | 2 | 1 | 5 | 6 | 3 | 7 | 5 | 11 | 3 | | | 1 | 64 |
| Gangrene | 2 | 1 | | 1 | 1 | | | | | 1 | | | | | | | | | 3 |
| Gout | 2 | 1 | 2 | | | | | | | | | | | | 1 | | | | 3 |
| Hemorrhage | 7 | 9 | 1 | 1 | | | 1 | | 2 | 3 | | 5 | 1 | 1 | 1 | | | | 16 |
| Inanition | 14 | 22 | 29 | | 2 | | | 1 | | 2 | 3 | | 1 | | | | | | 36 |
| Inflammation | 3 | 2 | 2 | 1 | | | | | | | | | | | | | | | 5 |
| " Glands | 2 | | 1 | | | 1 | | | | | | | | | | | | | 2 |
| " Throat | 4 | 1 | 2 | | 1 | | | | | | 1 | 1 | | | | | | | 5 |
| Malformation | 4 | 1 | 5 | | | | | | | | | | | | | | | | 5 |
| Marasmus | 122 | 116 | 141 | 69 | 17 | 2 | | | | 1 | 1 | 1 | | 5 | 1 | | | | 238 |
| Mortification | 1 | | | | | 1 | | | | | | | | | | | | | 1 |
| Scirrhus | 1 | 1 | | | | | | | | | | | 2 | | | | | | 2 |
| Scrofula | 9 | 13 | 5 | 7 | 3 | 3 | 2 | | 2 | | | | | | | | | | 22 |
| Sore Throat | 2 | 2 | 1 | | 2 | | 1 | | | | | | | | | | | | 4 |
| Tabes Mesenterica | 5 | 6 | 4 | 3 | 4 | | | | | | | | | | | | | | 11 |
| Tumor | 1 | | | 1 | | | | | | | | | | | | | | | 1 |
| Ulceration | 2 | 4 | | 1 | 1 | | | | 1 | | 1 | 1 | 1 | | | | | | 6 |
| " of Throat | 1 | 1 | | | 2 | | | | | | | | | | | | | | 2 |
| " of Leg | 1 | | | | | | | | | 1 | | | | | | | | | 1 |
| | 291 | 314 | 274 | 91 | 53 | 16 | 7 | 5 | 16 | 23 | 14 | 26 | 32 | 34 | 9 | 3 | 1 | 1 | 605 |

### 3. *Nervous Diseases.*

| | Male. | Female. | Under 1 yr. | 1 to 2. | 2 to 5. | 5 to 10. | 10 to 15. | 15 to 20. | 20 to 30. | 30 to 40. | 40 to 50. | 50 to 60. | 60 to 70. | 70 to 80. | 80 to 90. | 90 to 100. | 100 to 110. | Total. |
|---|---|---|---|---|---|---|---|---|---|---|---|---|---|---|---|---|---|---|
| Abscess of Brain | 1 | 1 | 1 | | | 1 | | | | | | | | | | | | 2 |
| Apoplexy | 11 | 19 | | | | | | | 2 | 5 | 4 | 7 | 5 | 6 | 1 | | | 30 |
| Coma | | 1 | | | | | | | | | 1 | | | | | | | 1 |
| Concussion of Brain | 1 | | 1 | | | | | | | | | | | | | | | 1 |
| Congestion " | 35 | 34 | 19 | 16 | 8 | 4 | 2 | 3 | 3 | 3 | 4 | 4 | 3 | | | | | 69 |
| Compression " | 4 | 1 | | | 1 | 1 | | | | | 1 | 1 | | 1 | | | | 5 |
| Convulsions | 103 | 78 | 103 | 40 | 18 | 6 | 1 | 3 | 4 | 3 | 2 | | | 1 | | | | 181 |
| Cramp | 1 | 1 | | | | | | | | | | 1 | 1 | | | | | 2 |
| Disease of Brain | 16 | 19 | 11 | 9 | 9 | | | 2 | 3 | | | 1 | | | | | | 35 |
| Dropsy " | 59 | 32 | 43 | 22 | 21 | 3 | | | 1 | 1 | | | | | | | | 91 |
| Effusion " | 20 | 7 | 6 | 10 | 5 | | 1 | 1 | 1 | 1 | | 1 | 1 | | | | | 27 |
| Epilepsy | 5 | 5 | 1 | | 1 | 1 | | | 1 | 3 | 1 | 2 | | | | | | 10 |
| Inflammation of Brain | 62 | 49 | 44 | 28 | 18 | 7 | 2 | 1 | 3 | 3 | | 4 | 1 | | | | | 111 |
| Mania | 1 | 1 | | | | | | | 2 | | | | | | | | | 2 |
| " a Potu | 9 | 1 | | | | | | | 2 | 5 | 3 | | | | | | | 10 |
| Neuralgia | 1 | | | | | | | | | | 1 | | | | | | | 1 |
| Palsy | 11 | 16 | | | 1 | | | | 2 | 3 | 2 | 3 | 10 | 3 | 1 | 2 | | 27 |
| Softening of Brain | 6 | 6 | | | | | | | 1 | 1 | 3 | 2 | 2 | 2 | 1 | | | 12 |
| Sun Stroke | 1 | | | | | | | 1 | | | | | | | | | | 1 |
| Tetanus | 4 | | | | | | | | 1 | 2 | | 1 | | | | | | 4 |
| Trismus | 1 | 1 | 2 | | | | | | | | | | | | | | | 2 |
| | 352 | 272 | 231 | 125 | 82 | 23 | 6 | 11 | 26 | 30 | 22 | 27 | 23 | 13 | 3 | 2 | | 624 |

### 4. *Organs of Respiration.*

| | Male. | Female. | Under 1 yr. | 1 to 2. | 2 to 5. | 5 to 10. | 10 to 15. | 15 to 20. | 20 to 30. | 30 to 40. | 40 to 50. | 50 to 60. | 60 to 70. | 70 to 80. | 80 to 90. | 90 to 100. | 100 to 110. | Total. |
|---|---|---|---|---|---|---|---|---|---|---|---|---|---|---|---|---|---|---|
| Abscess of Lungs | | 1 | | | 1 | | | | | | | | | | | | | 1 |
| Asthma | 4 | 3 | 1 | | 1 | | | | 1 | | 2 | | 1 | | 1 | | | 7 |
| Congestion of Lungs | 9 | 11 | 6 | 4 | 1 | 2 | 1 | | 2 | 1 | | 2 | | | 1 | | | 20 |
| Consumption " | 160 | 210 | 13 | 9 | 8 | 4 | 6 | 20 | 126 | 85 | 47 | 34 | 12 | 6 | | | | 370 |
| Disease of Chest | 1 | | | | | | | | | | | | | 1 | | | | 1 |
| " Lungs | 4 | 4 | | 1 | | 1 | | | | 2 | 1 | | 1 | 2 | | | | 8 |
| Dropsy of Chest | 5 | 5 | 1 | 2 | 1 | 1 | | | 1 | 1 | 1 | 1 | 1 | | | | | 10 |
| Effusion of Lungs | 1 | 1 | | | 1 | | | 1 | | | | | | | | | | 2 |
| Emphysema | 1 | | | | | | | | 1 | | | 1 | | | | | | 1 |
| Fever Hectic | 1 | | | | | | | | 3 | | 1 | | | | | | | 1 |
| Hemorrhage of Lungs | 2 | 2 | | | | | | | | | | | | | | | | 4 |
| Inflamm'n of Bronchi | 11 | 13 | 14 | 2 | | | | | 1 | 1 | 1 | | 3 | 1 | 1 | | | 24 |
| " Larynx | 2 | 1 | | | 1 | | | | | | 1 | | | 1 | | | | 3 |
| " Lungs | 29 | 29 | 16 | 8 | 18 | 1 | 2 | 2 | 1 | 2 | 1 | 2 | 3 | 1 | 1 | | | 58 |
| " Pleura | 1 | 1 | | | | | | | | | 1 | 1 | | | | | | 2 |
| " Trachea | 1 | 2 | | 1 | 2 | | | | | | | | | | | | | 3 |
| | 232 | 283 | 51 | 27 | 34 | 9 | 9 | 23 | 136 | 92 | 56 | 41 | 21 | 12 | 4 | | | 515 |

5. *Organs of Circulation.*

| | Male. | Female. | Under 1 yr. | 1 to 2. | 2 to 5. | 5 to 10. | 10 to 15. | 15 to 20. | 20 to 30. | 30 to 40. | 40 to 50. | 50 to 60. | 60 to 70. | 70 to 80. | 80 to 90. | 90 to 100. | 100 to 110. | Total. |
|---|---|---|---|---|---|---|---|---|---|---|---|---|---|---|---|---|---|---|
| Anæmia | 3 | 3 | 5 | | 1 | | | | | | | | | | | | | 6 |
| Aneurism | | 1 | | | | | | | | | | | | 1 | | | | 1 |
| " of Aorta | | 1 | | | | | | | | 1 | | | | | | | | 1 |
| Disease of Heart | 16 | 23 | 3 | 4 | 3 | 2 | 1 | 1 | 3 | 4 | 6 | 6 | 3 | 2 | 1 | | | 39 |
| Dropsy " | 1 | 2 | | | | | | | | | | 1 | | 1 | 1 | | | 3 |
| Enlargement of Heart | 3 | 1 | | | | | 1 | | 2 | | 1 | | | | | | | 4 |
| Inflammation of " | 1 | | | | | | | | | | | | 1 | | | | | 1 |
| | 24 | 31 | 8 | 4 | 4 | 2 | 2 | 1 | 5 | 5 | 7 | 7 | 4 | 4 | 2 | | | 55 |

6. *Digestive Organs.*

| | Male. | Female. | Under 1 yr. | 1 to 2. | 2 to 5. | 5 to 10. | 10 to 15. | 15 to 20. | 20 to 30. | 30 to 40. | 40 to 50. | 50 to 60. | 60 to 70. | 70 to 80. | 80 to 90. | 90 to 100. | 100 to 110. | Total. |
|---|---|---|---|---|---|---|---|---|---|---|---|---|---|---|---|---|---|---|
| Cancrum Oris | 2 | | | 1 | | 1 | | | | | | | | | | | | 2 |
| Carcinoma of Liver | 1 | | | | | | | | 1 | | | | | | | | | 1 |
| Colic | 4 | 3 | 2 | 1 | | | 1 | | 1 | | 1 | | | 1 | | | | 7 |
| Congestion of Liver | 1 | 2 | | | | | 1 | 1 | | | 1 | | | | | | | 3 |
| " Stom. & Bowels | 2 | | | | | 1 | | | | | | | 1 | | | | | 2 |
| Consumption of Bowels | | 1 | | | | | | | | 1 | | | | | | | | 1 |
| Disease of Liver | 5 | 7 | 1 | 1 | | 1 | | | 3 | 1 | 2 | 1 | | 1 | 1 | | | 12 |
| " Mouth | 1 | | 1 | | | | | | | | | | | | | | | 1 |
| " Stom. & Bowels | 5 | 3 | 3 | 2 | 1 | 1 | | | | | 1 | | | | | | | 8 |
| Dropsy, Abdominal | 1 | 3 | | | | | 1 | | | 1 | 2 | | | | | | | 4 |
| Enlargement of Spleen | | 1 | | | | | | | | | | | 1 | | | | | 1 |
| Hemorrhage of Stomach | 2 | | | | | | | | | 1 | | 1 | | | | | | 2 |
| " Bowels | 1 | | | | | | | | | 1 | | | | | | | | 1 |
| Hernia Strangulated | 1 | 4 | | | | | | | | 2 | 1 | | 1 | 1 | | | | 5 |
| Inflammat. Colon | 1 | | | | 1 | | | | | | | | | | | | | 1 |
| " Liver | 8 | 4 | | | 1 | | | 1 | 1 | 1 | 5 | 2 | | 1 | | | | 12 |
| " Peritoneum | 5 | 8 | 1 | | | 3 | | 1 | 5 | 1 | 1 | 1 | | | | | | 13 |
| " Stom. & Bowels | 25 | 24 | 15 | 6 | 5 | 2 | 1 | 1 | 7 | 2 | 4 | 2 | 2 | 2 | | | | 49 |
| Intussusception | 1 | 1 | | | | | | | | | 1 | | 1 | | | | | 2 |
| Jaundice | 4 | 3 | 4 | 1 | | 1 | | | | | 1 | | | | | | | 7 |
| Obstruction of Bowels | 2 | 1 | | 2 | | | | | | 1 | | | | | | | | 3 |
| Rupture of Liver | 1 | | | | | | | | | | | 1 | | | | | | 1 |
| Softening of Stomach | 1 | 3 | 3 | | 1 | | | | | | | | | | | | | 4 |
| Sore Mouth | | 1 | 1 | | | | | | | | | | | | | | | 1 |
| Stomatitis | | 1 | | | 1 | | | | | | | | | | | | | 1 |
| Teething | 10 | 5 | 6 | 8 | | 1 | | | | | | | | | | | | 15 |
| Ulceration of Bowels | 5 | 2 | | 1 | 1 | 2 | | | | 1 | 1 | | 1 | | | | | 7 |
| Worms | 1 | 1 | | | 2 | | | | | | | | | | | | | 2 |
| | 90 | 78 | 37 | 23 | 13 | 13 | 4 | 4 | 18 | 13 | 21 | 8 | 7 | 6 | 1 | | | 168 |

### 7. *Urinary Organs.*

| | Male. | Female. | Under 1 yr. | 1 to 2. | 2 to 5. | 5 to 10. | 10 to 15. | 15 to 20. | 20 to 30. | 30 to 40. | 40 to 50. | 50 to 60. | 60 to 70. | 70 to 80. | 80 to 90. | 90 to 100. | 100 to 110. | Total. |
|---|---|---|---|---|---|---|---|---|---|---|---|---|---|---|---|---|---|---|
| Diabetes | 2 | | | 1 | | | | | 1 | | | | | | | | | 2 |
| Disease of Kidneys | 2 | | | | | | | | | 1 | | 1 | | | | | | 2 |
| Inflammation of Bladder | | 1 | | | | | | | | | | 1 | | | | | | 1 |
| " Kidneys | 2 | 1 | | | | | | 1 | | | | 2 | | | | | | 3 |
| Suppression of Urine | 1 | | | | | | | | | | | | | 1 | | | | 1 |
| | 7 | 2 | | 1 | | | | 1 | 1 | 1 | | 4 | | 1 | | | | 9 |

### 8. *Organs of Generation.*

| | Male. | Female. | Under 1 yr. | 1 to 2. | 2 to 5. | 5 to 10. | 10 to 15. | 15 to 20. | 20 to 30. | 30 to 40. | 40 to 50. | 50 to 60. | 60 to 70. | 70 to 80. | 80 to 90. | 90 to 100. | 100 to 110. | Total. |
|---|---|---|---|---|---|---|---|---|---|---|---|---|---|---|---|---|---|---|
| Cancer of Uterus | | 1 | | | | | | | | | 1 | | | | | | | 1 |
| Child Bed | | 2 | | | | | | | 2 | | | | | | | | | 2 |
| Chlorosis | | 2 | | 1 | | | | 1 | | | | | | | | | | 2 |
| Convulsions Puerperal | | 1 | | | | | | | 1 | | | | | | | | | 1 |
| Disease of Uterus | | 1 | | | | | | | | 1 | | | | | | | | 1 |
| Fever Puerperal | | 15 | | | | | | 1 | 11 | 3 | | | | | | | | 15 |
| Hemorrhage of Uterus | | 1 | | | | | | | | | | 1 | | | | | | 1 |
| Inflammation of " | | 4 | | 1 | | | | | 2 | 1 | | | | | | | | 4 |
| | | 27 | | 2 | | | | 2 | 16 | 5 | 1 | 1 | | | | | | 27 |

### 9. *Organs of Locomotion.*

| | Male. | Female. | Under 1 yr. | 1 to 2. | 2 to 5. | 5 to10. | 10 to 15. | 15 to 20. | 20 to 30. | 30 to 40. | 40 to 50. | 50 to 60. | 60 to 70. | 70 to 80. | 80 to 90. | 90 to 100. | 100 to 110. | Total. |
|---|---|---|---|---|---|---|---|---|---|---|---|---|---|---|---|---|---|---|
| Caries | | 1 | | 1 | | | | | | 1 | | | | | | | | 1 |
| Disease of Hip | 1 | 1 | | | | | 1 | | | | | | | | | | | 2 |
| " Spine | 1 | | | | | 1 | | | | | | | | | | | | 1 |
| Rheumatism | 1 | 2 | | | | | | | | 2 | | | 1 | | | | | 3 |
| | 3 | 4 | | 1 | | 1 | 1 | | | 3 | | | 1 | | | | | 7 |

10. *Integumentary System.*

| | Male. | Female. | Under 1 yr. | 1 to 2. | 2 to 5. | 5 to 10. | 10 to 15. | 15 to 20. | 20 to 30. | 30 to 40. | 40 to 50. | 50 to 60. | 60 to 70. | 70 to 80. | 80 to 90. | 90 to 100. | 100 to 110. | Total. |
|---|---|---|---|---|---|---|---|---|---|---|---|---|---|---|---|---|---|---|
| Malignant Pustule | 1 | | | | | | | | 1 | | | | | | | | | 1 |
| Purpura | 1 | | 1 | | | | | | | | | | | | | | | 1 |
| " Hemorrhagica | 4 | 3 | 5 | | | 1 | | | 1 | | | | | | | | | 7 |
| Scurvy | 2 | | | | | | | | | | | 1 | 1 | | | | | 2 |
| | 8 | 3 | 6 | | | 1 | | | 2 | | | 1 | 1 | | | | | 11 |

11. *Old Age.*

| | Male. | Female. | Under 1 yr. | 1 to 2. | 2 to 5. | 5 to 10. | 10 to 15. | 15 to 20. | 20 to 30. | 30 to 40. | 40 to 50. | 50 to 60. | 60 to 70. | 70 to 80. | 80 to 90. | 90 to 100. | 100 to 110. | Total. |
|---|---|---|---|---|---|---|---|---|---|---|---|---|---|---|---|---|---|---|
| Old Age | 9 | 27 | | | | | | | | | | | | 14 | 14 | 8 | | 36 |
| Still Born | 78 | 61 | 139 | | | | | | | | | | | | | | | 139 |
| Unknown | 50 | 29 | 31 | 3 | 8 | 1 | | 3 | 9 | 9 | 6 | 6 | 2 | 1 | | | | 79 |

12. *External Causes.*

| | Male. | Female. | Under 1 yr. | 1 to 2. | 2 to 5. | 5 to 10. | 10 to 15. | 15 to 20. | 20 to 30. | 30 to 40. | 40 to 50. | 50 to 60. | 60 to 70. | 70 to 80. | 80 to 90. | 90 to 100. | 100 to 110. | Total. |
|---|---|---|---|---|---|---|---|---|---|---|---|---|---|---|---|---|---|---|
| Asphyxia | 5 | 1 | 5 | | | 1 | | | | | | | | | | | | 6 |
| Burns | 4 | 7 | | 2 | 2 | 1 | 1 | 2 | 2 | | 1 | | | | | | | 11 |
| Casualties | 49 | 10 | | | 1 | | 14 | 14 | 13 | 9 | 4 | 3 | 1 | | | | | 59 |
| Drowned | 49 | 10 | | 1 | 3 | 12 | 7 | 6 | 11 | 11 | 5 | 2 | 1 | | | | | 59 |
| Exhaustion | 2 | 4 | 1 | 1 | | | | | 1 | 2 | 1 | | | | | | | 6 |
| Fracture | 4 | 1 | | | | | | | 1 | 2 | 1 | | | 1 | | | | 5 |
| " Skull | 1 | | | | | | | | | 1 | | | | | | | | 1 |
| Heat | 2 | | 1 | | | | | | | 1 | | | | | | | | 2 |
| Hydrophobia | 1 | | | | | | | | | 1 | | | | | | | | 1 |
| Intemperance | 3 | 5 | | | | | | | 4 | 1 | 3 | | | | | | | 8 |
| Poisoning | 1 | 1 | | | | | | | | 1 | | 1 | | | | | | 2 |
| Starvation | | 1 | 1 | | | | | | | | | | | | | | | 1 |
| Strangulation | 1 | | | | | | | | | | | | | 1 | | | | 1 |
| Suffocation | 1 | | 1 | | | | | | | | | | | | | | | 1 |
| Suicide | 9 | 2 | | | | | | | 3 | 2 | 2 | | 4 | | | | | 11 |
| | 132 | 42 | 9 | 4 | 6 | 14 | 22 | 22 | 35 | 31 | 17 | 6 | 6 | 2 | | | | 174 |

TABLE NO. 3.

*Deaths for the Third Quarter of* 1856, *at fifteen distinct periods of life.*

| | |
|---|---|
| Under 1 year, | 1246 |
| 1 to 2 | 642 |
| 2 to 5 | 421 |
| 5 to 10 | 167 |
| 10 to 15 | 74 |
| 15 to 20 | 96 |
| 20 to 30 | 329 |
| 30 to 40 | 242 |
| 40 to 50 | 179 |
| 50 to 60 | 154 |
| 60 to 70 | 115 |
| 70 to 80 | 111 |
| 80 to 90 | 39 |
| 90 to 100 | 15 |
| 100 to 110 | 1 |
| 110 to 120 | 1 |
| | 3832 |
| Still Born | 139 |
| Total, | 3971 |

Included in the above table of deaths, there were 185 from the Blockley Alms House; from among the colored population, 187; from the country, 47; the County Prison, 6; the Eastern Penitentiary, 1; and 24 from the Pennsylvania Hospital, as follows:—

| | July. | Aug. | Sept. | Total. |
|---|---|---|---|---|
| Almshouse, | 80 | 60 | 45 | 185 |
| Blacks, | 87 | 62 | 38 | 187 |
| Country, | 15 | 17 | 15 | 47 |
| Pennsylvania Hospital, | 13 | 6 | 3 | 22 |
| County Prison, | | | 6 | 6 |
| Eastern Penitentiary, | | 1 | | 1 |
| | 195 | 146 | 107 | 448 |

The following table gives the number of deaths for each week, during the quarter, together with the sexes, the adults, and children or those under twenty years of age:

| 1856. | Male. | Female. | Adults. | Children. | Total. | Mon. total. |
|---|---|---|---|---|---|---|
| From June 28th to July 5th, | 180 | 152 | 98 | 234 | 332 | |
| " July 5th to July 12th, | 186 | 143 | 93 | 236 | 329 | |
| " July 12th to July 19th, | 176 | 153 | 102 | 227 | 329 | |
| " July 19th to July 26th, | 221 | 205 | 104 | 322 | 426 | |
| " July 26th to Aug, 2d, | 195 | 185 | 95 | 285 | 380 | 1796 |
| " Aug. 2d to Aug. 9th, | 219 | 186 | 94 | 311 | 405 | |
| " Aug. 9th to Aug. 16th, | 175 | 179 | 117 | 237 | 354 | |
| " Aug. 16th to Aug. 23d, | 158 | 130 | 88 | 200 | 288 | |
| " Aug. 23d to Aug. 30th, | 144 | 120 | 79 | 185 | 264 | 1311 |
| " Aug. 30th to Sept. 6th, | 134 | 116 | 78 | 172 | 250 | |
| " Sept. 6th to Sept. 13th, | 120 | 105 | 84 | 141 | 225 | |
| " Sept. 13th to Sept. 20th, | 94 | 91 | 80 | 105 | 185 | |
| " Sept. 20th to Sept. 27th, | 103 | 101 | 75 | 129 | 204 | 864 |
| Total, | 2105 | 1866 | 1187 | 2784 | 3971 | 3971 |

